# Improving wheelchair user sitting posture to alleviate lumbar fatigue: a study utilizing sEMG and pressure sensors

**DOI:** 10.3389/fnins.2024.1380150

**Published:** 2024-03-15

**Authors:** Zizheng Huang, Jianwei Cui, Yuanbo Wang, Siji Yu

**Affiliations:** Jiangsu Key Lab of Remote Measurement and Control, School of Instrument Science and Engineering, Southeast University, Nanjing, China

**Keywords:** sEMG, muscle fatigue, MPF, sitting posture detection, multiple nonlinear regression

## Abstract

**Background:**

The wheelchair is a widely used rehabilitation device, which is indispensable for people with limited mobility. In the process of using a wheelchair, they often face the situation of sitting for a long time, which is easy to cause fatigue of the waist muscles of the user. Therefore, this paper hopes to provide more scientific guidance and suggestions for the daily use of wheelchairs by studying the relationship between the development of muscle fatigue and sitting posture.

**Methods:**

First, we collected surface Electromyography (sEMG) of human vertical spine muscle and analyzed it in the frequency domain. The obtained Mean Power Frequency (MPF) was used as the dependent variable. Then, the pose information of the human body, including the percentage of pressure points, span, and center of mass as independent variables, was collected by the array of thin film pressure sensors, and analyzed by a multivariate nonlinear regression model.

**Results:**

When the centroid row coordinate of the cushion pressure point is about 16(range, 7.7-16.9), the cushion pressure area percentage is about 80%(range, 70.8%-89.7%), and the cushion pressure span range is about 27(range, 25-31), the backrest pressure point centroid row coordinate is about 15(range, 9.1-18.2), the backrest pressure area percentage is about 35%(range, 11.8%-38.7%), and the backrest pressure span range is about 16(range, 9-22). At this time, the MPF value of the subjects decreased by a small percentage, and the fatigue development of the muscles was slower. In addition, the pressure area percentage at the seat cushion is a more sensitive independent variable, too large or too small pressure area percentage will easily cause lumbar muscle fatigue.

**Conclusion:**

The results show that people should sit in the middle and back of the seat cushion when riding the wheelchair, so that the Angle of the hip joint can be in a natural state, and the thigh should fully contact the seat cushion to avoid the weight of the body concentrated on the buttocks; The back should be fully in contact with the back of the wheelchair to reduce the burden on the waist, and the spine posture can be adjusted appropriately according to personal habits, but it is necessary to avoid maintaining a chest sitting position for a long time, which will cause the lumbar spine to be in an unnatural physiological Angle and easily lead to fatigue of the waist muscles.

## 1 Introduction

With the spread of electronic communication devices and changes in office practices, sedentary lifestyles are spreading around the world, with approximately one-third of the global population aged 15 years and older experiencing inactivity (Park et al., 2020). Unlike the sedentary daily office population, wheelchair users are forced to sit in wheelchairs for long periods due to their lack of autonomous mobility.

In modern society, wheelchairs, as an important rehabilitation aid, are widely used in individuals with limited mobility, especially those suffering from spinal cord injury, motor nervous system diseases, elderly people, or other lower limb mobility disorders (Mikołajewska and Mikołajewski, 2013; Leaman and La, 2017). Although wheelchairs play an important role in improving the quality of life and social participation of these people, the health problems caused by prolonged use of wheelchairs also require attention. When people are in a sitting posture, the lumbar muscle group plays a key role in supporting the spine and maintaining posture stability (Callaghan and McGill, 2001; O’Sullivan et al., 2002; Nowakowska et al., 2017), and is an important part of the core muscle group of the human body. However, sitting in a wheelchair for a long time may lead to persistent tension and fatigue of the waist muscles, which may cause discomfort in the waist, which greatly affects the comfort of wheelchair users.

Previous studies have pointed out that different sitting positions have a direct impact on waist comfort (Vergara and Page, 2002). One study (Black et al., 1996) classified sitting positions as comfortable (taking a comfortable, relaxed position), hunched, upright, and forward-leaning (leaning forward by hip flexion), and evaluated the effect of lumbar and pelvic tilt changes (achieved by hip flexion) on cervical vertebrae while maintaining lumbar lordosis. Another study (Claus et al., 2009) classified the Slump, Flat, Long lordosis, and Short lordosis types of sitting based on their thoracolumbar and lumbar angles, and discussed which spinal formations were beneficial. The methods adopted in these studies need to verbally describe or show pictures to the subjects to make them imitate each posture. The postures presented vary greatly under the influence of people’s subjective factors, so the detection and classification of sitting posture require more objective features or indicators.

There are also differences in people’s sitting posture in different scenarios. Current studies on sitting comfort in different scenarios include: Literature (Schmidt et al., 2014) analyzed several papers suggesting the optimal driving posture of cars, and discussed several factors affecting the optimal posture such as gender, seat design, body shape, and age; Literature (Mörl and Bradl, 2013) studied the waist posture and muscle activity of the human body in the office. However, most people who use wheelchairs lack self-care ability and often face a situation in which they are in a relatively fixed posture for dozens of minutes or even longer, so a reasonable sitting posture will be very effective in delaying the generation of lumbar muscle fatigue of wheelchair users. At the muscle level, fatigue means that muscle fibers have a reduced ability to produce force (Marco et al., 2017). The measurement of fatigue in the state of exercise is generally to measure the change of muscle power or power, which is achieved by the maximum autonomous contraction test (MVCs) (Yousif et al., 2019). For wheelchair users, most of the time the body is in a static state, so the use of non-invasive technology such as sEMG can better assess the state of muscle fatigue (Barsotti et al., 2020). For example, literature (Liu et al., 2019) studies an EMG patch device that can be transmitted wirelessly and uses the EMD method to decompose the sEMG signal to obtain MF value to evaluate muscle fatigue state. In another paper (Cahyadi et al., 2019), DELSYS Bagnoli EMG 8 channel and surface EMG sensor-based single difference system were used to collect sEMG data, and the average power frequency (MNF) in frequency domain analysis was used as an index to study the progress of muscle fatigue during arm movement.

At present, there are relatively few studies on the influence of different sitting positions on lumbar fatigue during wheelchair use. Therefore, the objective of this paper is to obtain the lumbar muscle fatigue status of wheelchair users by studying the detection method of human sitting position and sMEG collection and analysis method and to combine the characteristics of human sitting position with frequency domain analysis. The effects of different sitting conditions on the fatigue progress of lumbar muscles were analyzed. Ultimately, we hope to provide more scientific recommendations for wheelchair users by comparing the effects of different sitting positions on lumbar muscle fatigue.

The remainder of this article is organized as follows. In Section 2, we describe the materials and methods used in the study. In Section 3, we describe the steps of the experiment and the multivariate nonlinear regression model. In Section 4, we present the results and discuss them. Finally, we give a conclusion about the whole article.

## 2 Materials and methods

### 2.1 sEMG data acquisition methods

**Figure 1:**
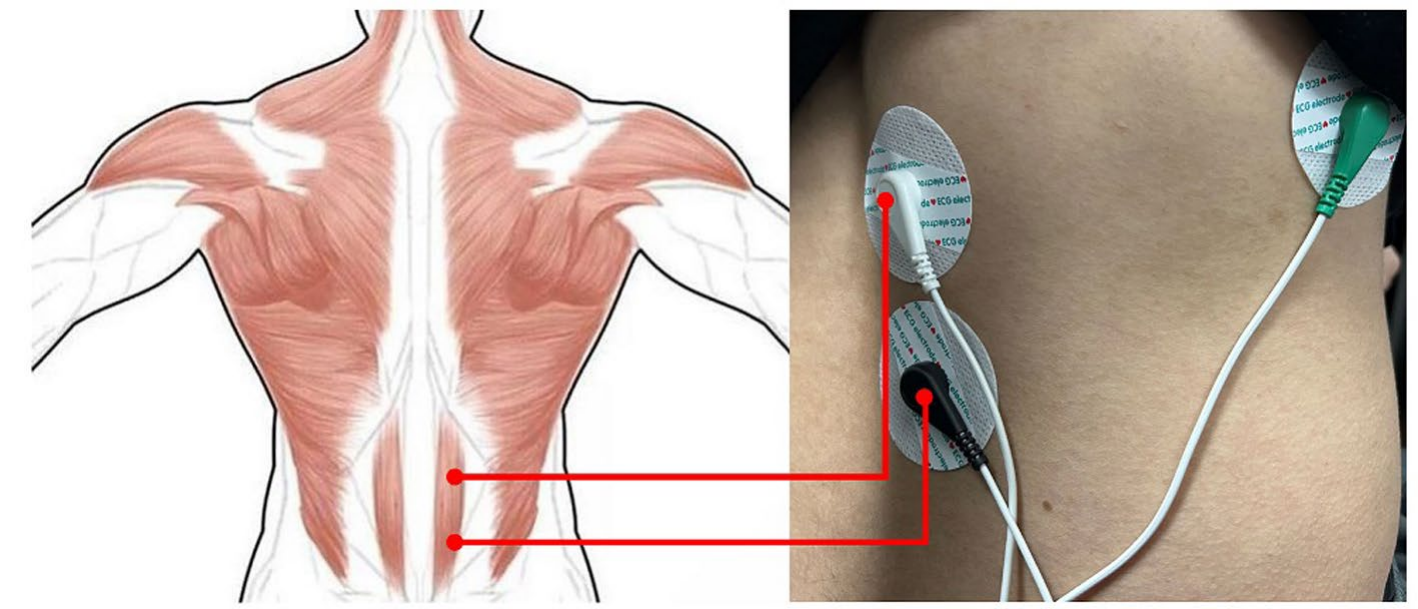
Schematic diagram of Ag/AgCl electrode placement.

The sEMG signals collection process for muscle fatigue analysis in this study is as follows: Initially, sEMG signals raw signals were obtained from the erector spinae muscles using Ag/AgCI electrodes with adhesive properties and wires (as shown in Figure 1, where white represents positive, black represents negative, and green represents the reference electrode). The wires were connected to an sEMG signals acquisition board for signal collection and storage, and finally, the data were imported to a PC for further analysis.

**Figure 2:**
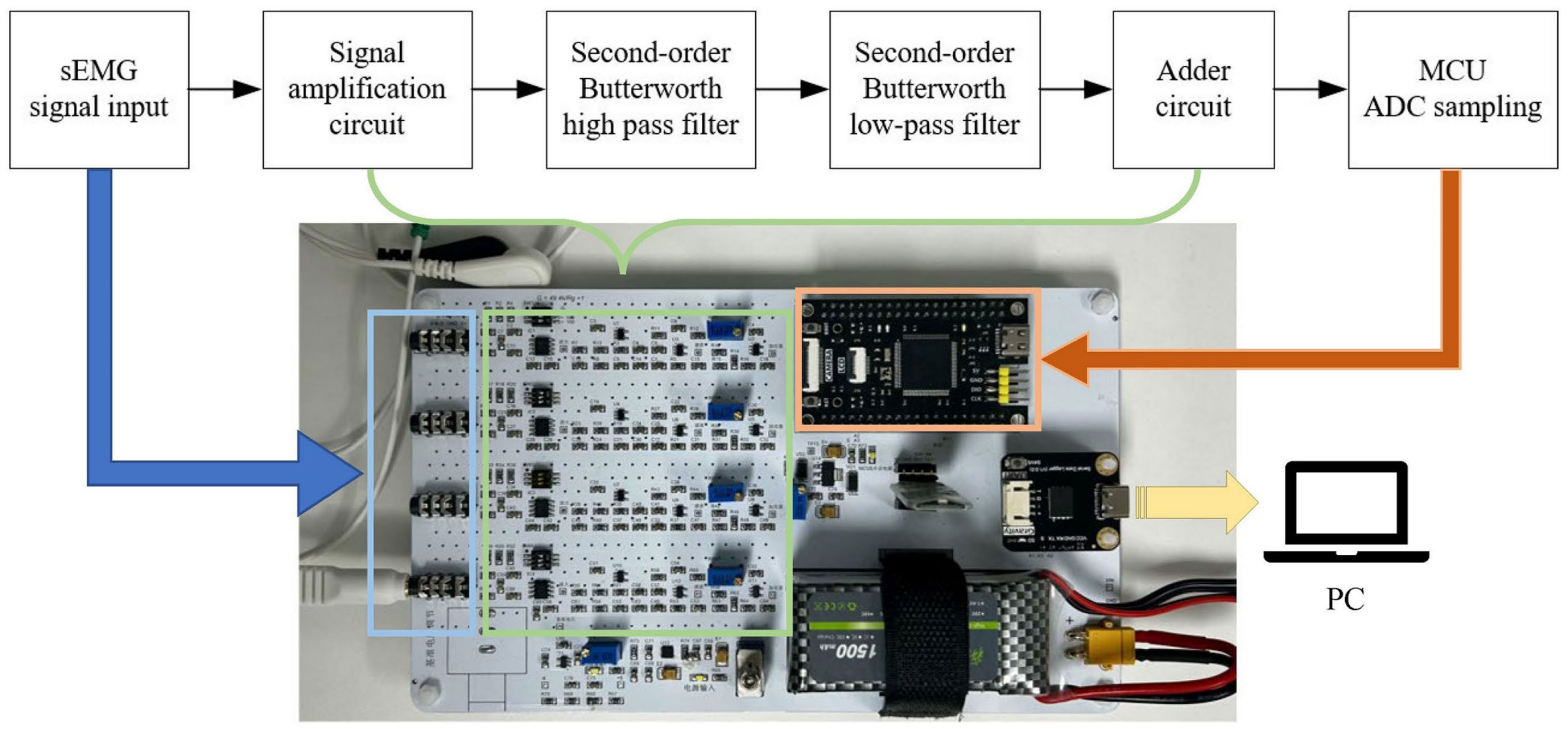
sEMG acquisition board structure diagram.

The sEMG signals acquisition board, designed and manufactured by our team, is depicted in Figure 2. According to the Nyquist sampling theorem, the sampling frequency must be twice the maximum signal frequency. As the frequency range of sEMG signals is 0 ~ 500 Hz, the sampling rate of our designed sEMG signals acquisition board is set at 1000 Hz.Since the original sEMG signal is a weak electrical signal with a small amplitude that is challenging to directly collect, we initially used an amplifier circuit with a gain of 500 (AD620, Analog Devices) to amplify it. Subsequently, the signal underwent filtering processing through a second-order Butterworth high-pass filter with a cutoff frequency of 10 Hz and a second-order Butterworth low-pass filter with a cutoff frequency of 500 Hz. This filtering aimed to avoid interference from DC components, baseline drift, and high-frequency signals. Since the ADC (Analog-to-Digital Converter) of the microcontroller can only accept non-negative inputs, and the amplitude of the electromyographic signal is negative, the signal also needs to be elevated through an adder circuit (LM321, Texas Instruments). Finally, we collected the sEMG signal through the ADC pins of the microcontroller and stored it in memory for retrieval by the PC.

### 2.2 Data preprocessing

The raw sEMG data collected after acquisition is in the form of digital output from the ADC. Therefore, we need to perform a calculation to restore the amplitude of the signal. The calculation formula is shown in Equation 1.


(1) $$\mathrm{sEMG}\;\mathrm{Amplitude}=\frac{\mathrm{Digital}\;\mathrm{value}.V_{ref}\;}{2^{n}-1}-V_{\mathrm{add}}$$

**Figure 3:**
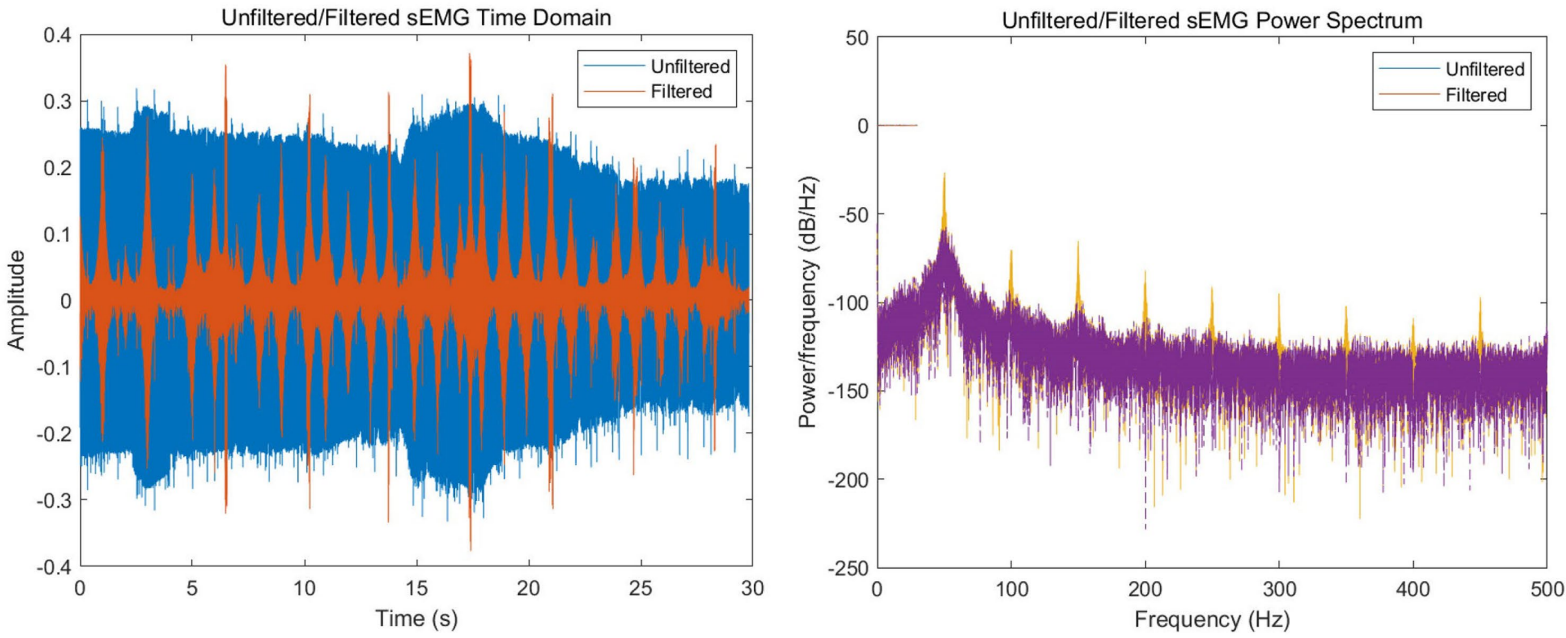
Time-domain and power spectral density comparison of sEMG signals before and after notch filtering.

In the formula, Digitalvalue is the digital value output by the ADC, $V_{ref}$ is the reference voltage of the ADC, n is the number of bits of the ADC, and $V_{\mathrm{add}}$ is the elevated voltage value of the adder circuit. The raw sEMG data is already in a relatively suitable frequency range through the filter in the circuit, but we also need to filter it to eliminate power frequency interference. In Figure 3, we can see from the power density diagram of the original signal that there are relatively obvious power frequency interference of 50 Hz and its harmonics, so we use a 45 dB attenuated Butterworth trap filter in Matlab to eliminate these noises.

### 2.3 Muscle fatigue analysis methods

After the collection of sEMG data is completed, frequency domain analysis of sEMG data is required to obtain characteristic parameters related to the development of muscle fatigue. Among them, MF median frequency and MPF average power frequency are commonly used to judge muscle fatigue (Cao et al., 2018). The average power frequency is the weighted average of the spectrum, obtained by multiplying each frequency component of the spectrum by its corresponding power, then summing all frequencies, and finally dividing by the total power. The basic formula is shown in Equation 2.


(2) $$MPF=\frac{\int_{f_{a}}^{f_{b}}f.P\left(f\right)df}{\int_{f_{a}}^{f_{b}}P\left(f\right)df}$$

In the formula, f represents frequency, $f_{a}$ and $f_{b}$ are the upper and lower limits of the spectrum, and $P\left(f\right)$ is the power at the frequency f .

### 2.4 Sitting posture detection methods

As for the detection methods of human posture, scholars from various countries have proposed a variety of feasible schemes. For example, van Nassau et al. (2015) and Rowlands et al. (2014) chose the scheme of activPAL activity monitor, which detected human posture by wearing an accelerometer on the wrist and thigh of the subjects. However, this method can only roughly determine whether the human body is in a standing or sitting position, and cannot obtain more detailed information about the human posture. Estrada and Vea (2016) used a customized belt to fix three gyroscopes on the subjects’ thoracic vertebrae, thoracolumbar vertebrae, and lumbar vertebrae to detect the human sitting position. Although this scheme could better restore the shape of the human spine, due to the need for additional wearing devices on the back during the detection process, it could not fully restore the natural sitting position. Similar to the scheme adopted by Huang and Ouyang (2012) and Matuska et al. (2020), multiple pressure sensors were installed on the back and cushion of the seat to detect the human sitting position. However, due to the small number of sensor points, this scheme could only detect several preset sitting types.

**Figure 4:**
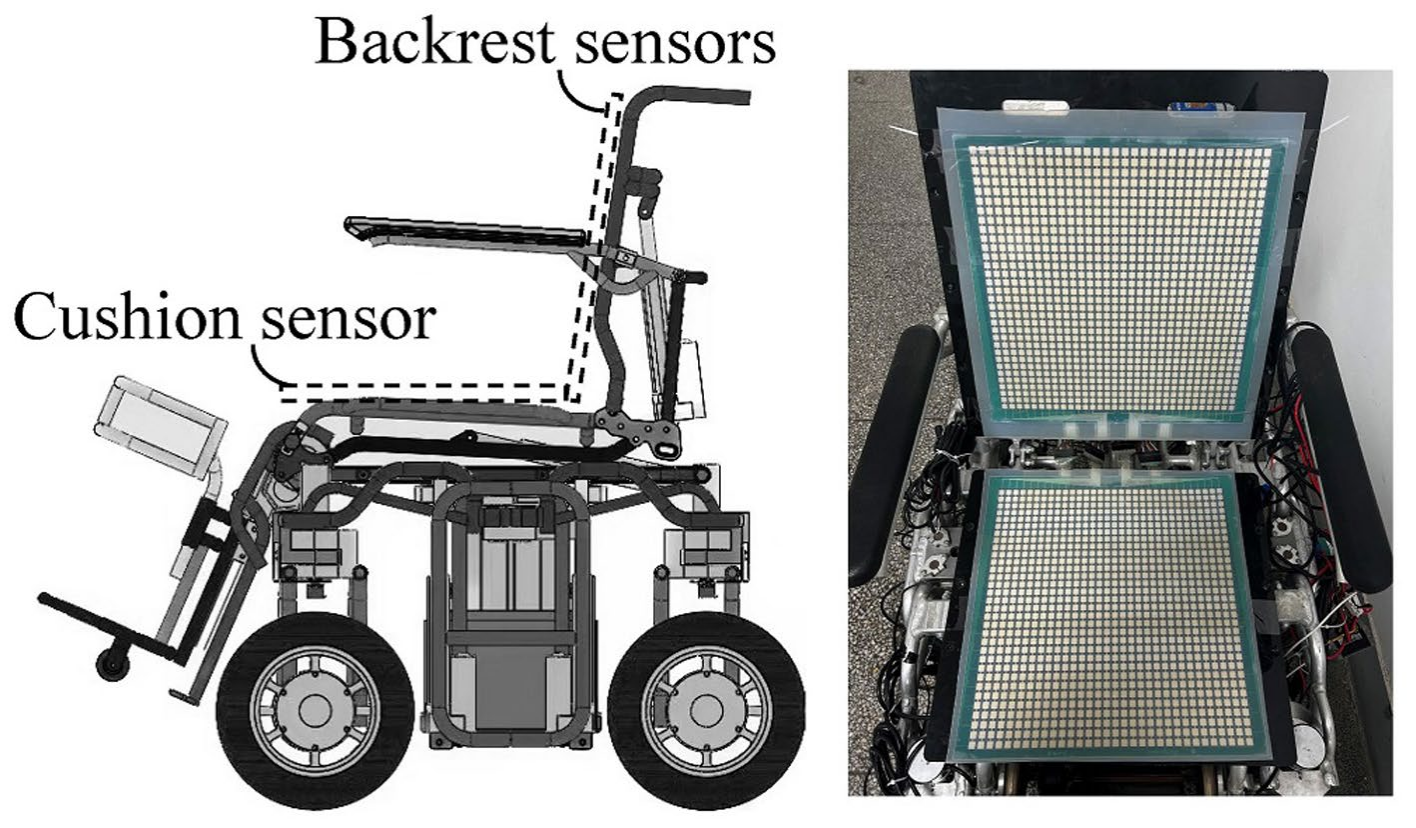
Schematic diagram of thin film pressure sensor installation locations.

In the actual sitting state, due to the differences in people’s sitting habits and body types, it is not completely accurate to distinguish the sitting position of the human body simply by the Angle of the seat. Therefore, to better obtain the force distribution of the wheelchair without affecting the seated position of the passenger, a 32*32 array thin film pressure sensor was selected to be installed on the cushion and back of the wheelchair, as shown in Figure 4. This array pressure sensor has 1,024 independent sampling points. It can better restore the force of the back of the wheelchair and the cushion, and use it to distinguish the sitting situation of the human body. According to the actual sampling requirements, our team independently designed a collection card system (Cui et al., 2023), which can collect and report data for each point.

### 2.5 Sitting posture feature extraction methods

Seating posture data is obtained from the array-type thin-film pressure sensor mentioned in Section 2.4. Each force unit of this sensor acts as a variable resistor. Based on this characteristic, we designed a data acquisition card with a voltage series negative feedback amplifier circuit, capable of collecting the resistance values at each point on the sensor. Additionally, the acquisition card features a row-column scanning circuit, which, under MCU control, connects the specified force unit to the feedback amplifier circuit, followed by voltage value collection through ADC. According to the characteristics of the series negative feedback amplifier circuit, the output voltage of the ADC can be derived from Equation 3.


(3) $$V_{\mathrm{out}}=\left(1+\frac{R_{f}\;}{R}\right)\times V_{\mathrm{in}}$$

**Figure 5:**
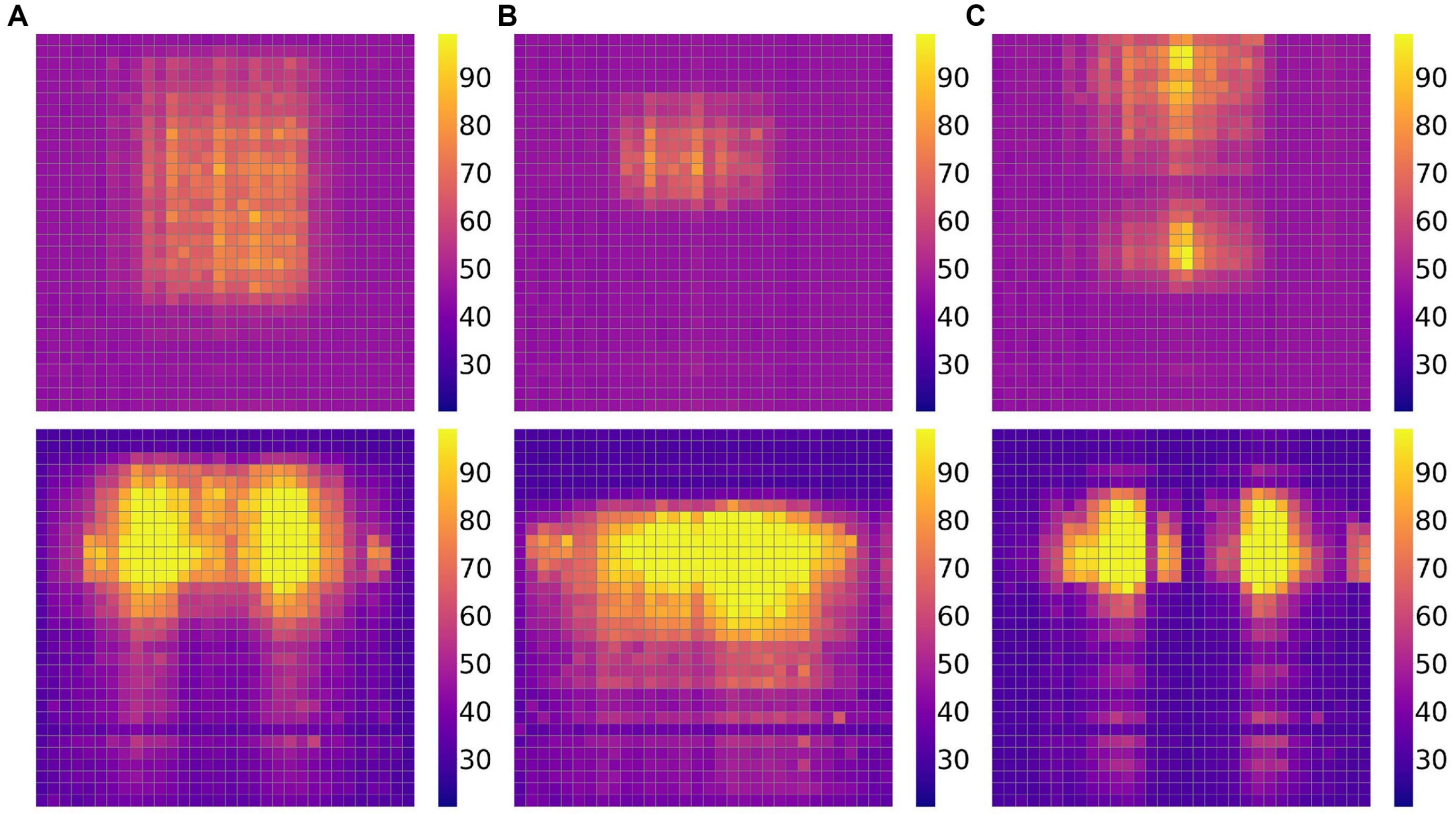
Three sets of pressure data graphs for different situations, with the upper part being the backrest and the lower part being the seat cushion: Part **(A)** represents the seating position being rear; Part **(B)** indicates that the seating position is in the front; Part **(C)** indicates a moderate seating position.

In the formula, $R_{f}$ represents the resistance value of the force unit, R is a fixed resistor, $V_{\mathrm{in}}$ is the input voltage at the inverting terminal of the operational amplifier. Therefore, through the ADC-collected voltage value $V_{\mathrm{out}}$ , we can obtain the value of $R_{f}$ . Then, using the characteristic curve of $R_{f}$ with pressure, the pressure received by each force unit can be calculated. For the collected pressure data, we will save it through the upper computer of the PC. The pressure data of several different sitting states are shown in Figure 5. Part A shows a sitting position with a relatively backward seating position and a large backrest contact area; Part B demonstrates a sitting position with a relatively forward seating position and a small backrest contact area; Part C demonstrates a sitting posture in which the seating position and backrest contact area are moderate.

**Figure 6:**
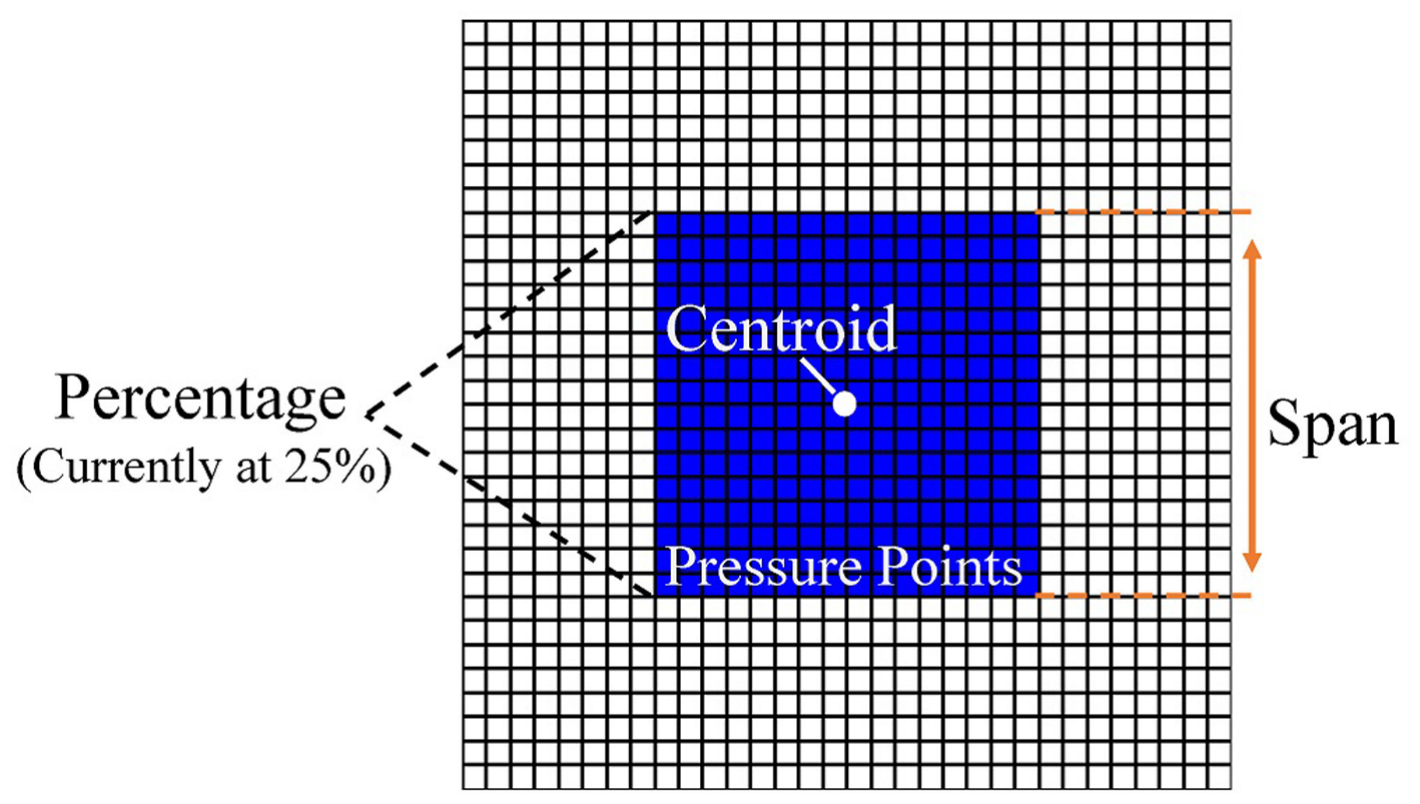
Schematic diagram of three seating posture features.

For the collected pressure data, we extract three features to reflect the body posture: pressure percentage, span, and centroid, as shown in Figure 6. The percentage refers to the percentage of pressure points exceeding a certain threshold in the entire sitting posture area compared to the total number of sensor force units. A higher percentage indicates a larger contact area between the body and the wheelchair, reflecting a more even pressure distribution, while a lower percentage indicates a smaller contact area. The span represents the distribution range of pressure points in the row direction, i.e., the difference between the top and bottom rows of pressure points, reflecting the variation range of the sitting posture in the row direction. The centroid represents the average position of pressure points in the entire sitting posture. It is the center position calculated based on weights (i.e., pressure values). Here, we only calculate the row coordinate of the centroid (the column coordinate is more correlated with the occupant’s position relative to the sensor and less relevant to the sitting posture), and its calculation formula is shown in Equation 4.


(4) $$\mathrm{Centroid}:\mathrm{row}=\frac{\sum_{i}y_{i}p_{i}}{\sum_{i}y_{i}}$$

In the formula, $y_{i}$ represents the vertical coordinate of the pressure point, and $p_{i}$ represents the pressure value of that point. The above three characteristic quantities provide information about the distribution, balance, and range of variation of sitting posture respectively, and also include body shape factors to a certain extent. Compared with the human posture features such as lumbar spine and pelvic Angle commonly used in previous studies (Ma et al., 2016; Wan et al., 2021), the features derived from pressure data can more truly reflect the load distribution and posture changes of the human body on the wheelchair, rather than the information of some specific joints or parts. Moreover, there is no need to wear additional equipment in the collection process. The error caused by flexible materials such as clothing and straps can be eliminated, and the sitting position of the human body can be better restored and presented.

## 3 Experiment and analysis

### 3.1 Experiment procedures

The experiment invited 20 male volunteers, aged between 21 and 34, whose daily work and life were mainly office environment. All volunteers had no diseases related to the waist or spine. We informed the subjects about the types and uses of data to be collected and obtained their consent. Before the experiment, we will inform each subject of the specific process of the experiment, including the placement of electrode strips and the process of data collection. To minimize the impact on the subject’s wheelchair usage habits, the sitting posture in the wheelchair is not specified, and personal usage habits shall be subject to.

**Figure 7:**
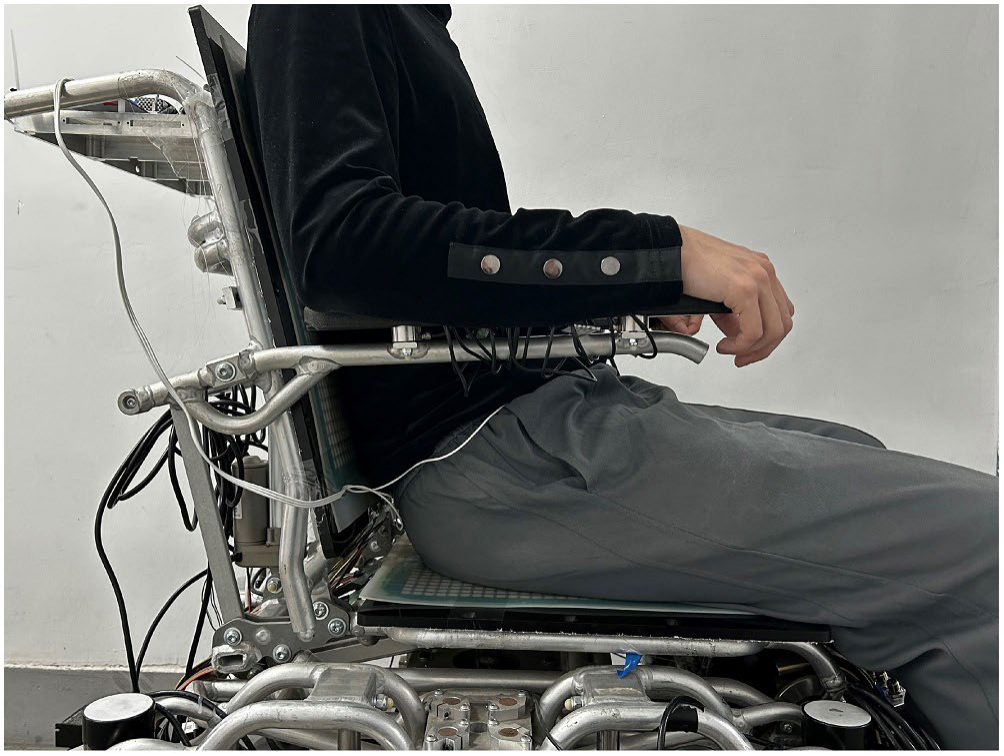
Schematic Diagram of muscle fatigue and seating posture detection experiment.

To ensure that people’s physical state is relatively consistent, the experiment is chosen to be carried out at 9 o ‘clock every day, and only one experiment is carried out every day. Before the formal start of each experiment, we first disinfected and cleaned the waist skin of the subjects, and then placed the electrode patch at the position of the vertical spinal muscle and started the collection of sEMG. When the subject sits in the wheelchair, we will properly adjust the leg support Angle of the wheelchair so that the knee joint is in a state of 90°, and the foot height will be adjusted so that each subject’s feet can be naturally placed on the foot without hanging. The backrest Angle and foot Angle of the wheelchair remain unchanged, and the volunteers can freely adjust their sitting position within 1 min after the start of the experiment until the most comfortable state, as shown in Figure 7. The whole experiment will last for 1 h. During the experiment, the fit degree between the back of the subject and the back of the wheelchair should be kept unchanged as far as possible. The buttocks and legs should not have large movements, while the upper limbs can move freely. We will check the current sitting position through the upper computer every 5 min to ensure that there is no major change in sitting position.

We will collect the sitting position data and the sEMG data of the vertical spine muscle for the first time 1 min after the start of the experiment. When the experiment time reaches 60 min, we will collect the sEMG data of the vertical spine muscle for the second time, and the duration of each sEMG data collection is 30 s.

### 3.2 Multiple nonlinear regression

In all the experimental data, the dependent variable was the percentage decrease in the average power frequency of the EMG signal, and the independent variable was six sitting features from the human body. Due to the non-simple linear relationship between the dependent variable and independent variables in this study, the data exhibits different curvatures in certain regions. Therefore, a multivariate nonlinear regression analysis is employed to analyze the relationship between fatigue values and sitting posture features. In multivariate nonlinear regression, it is commonly assumed that there is a complex nonlinear relationship between the dependent and independent variables, and nonlinear functions are used to fit the data. This model can more accurately describe the relationship between actual data and variables, capturing nonlinear effects.

We used MATLAB software for data analysis and multivariate binomial regression toolbox for regression analysis. The basic formula of the pure quadratic model invoked is shown in Equation 5.


(5) $$y=\left(\beta_{0}+\beta_{1}x_{1}+\ldots +\beta_{m}x_{m}+\overset{m}{\underset{i=1}{\sum }}\beta_{ii}x_{i}^{2}\right)$$

In the equation, y is the dependent variable, $x_{1}-x_{m}$ are independent variables, $\beta_{0}$ is the intercept, $\beta_{0}-\beta_{m}$ are coefficients of independent variables, and $\beta_{11}-\beta_{\mathrm{mm}}$ are coefficients of quadratic terms. The experimental data are shown in Table 1. The leftmost part is the dependent variable, that is, the percentage decline of MPF value, and the rest is the independent variable. From left to right, they are respectively: line coordinates of the centroid of the pressure point at the cushion, percentage of the pressure area at the cushion, row span of the pressure area at the cushion, line coordinates of the centroid of the pressure point at the backrest, percentage of the pressure area at the backrest and row span of the pressure at the backrest.

**Table 1:** Experimental data.

MPF	Seat			Backrest		
	Centroid	Percentage	Span	Centroid	Percentage	Span
−6.2894	15.30519	82.48828	28	13.2854	38.76953	22
−6.9056	12.77845	76.95313	30	11.51089	30.56641	21
−6.4382	13.82326	83.88672	28	12.81855	31.93359	16
−7.2153	10.03658	70.85938	28	9.18744	37.20703	22
−6.9228	11.05657	75.76953	26	10.90965	18.75	14
−6.321	16.49812	80.53906	26	14.48153	29.88281	20
−6.5555	13.20405	78.61328	28	14.27513	31.54297	20
−7.1298	7.769629	89.78906	29	16.97433	32.03125	14
−6.8112	9.52833	77.27344	26	16.53466	11.81641	9
−6.7243	14.36115	77.12891	23	18.23038	22.16797	17
−6.5408	9.067584	81.05469	28	14.7101	27.05078	21
−7.0093	10.89296	86.64063	28	16.17976	33.39844	22
−6.7687	12.06241	80.85938	30	15.37197	32.51953	21
−6.5976	14.04636	79.48828	28	12.22669	22.94922	17
−6.4891	16.96319	80.61328	25	10.25274	31.64063	15
−7.0564	11.3105	72.36328	26	15.07457	15.82031	14
−6.6645	14.0427	78.61328	29	9.301891	26.75781	21
−6.8461	10.63302	85.21484	31	15.36672	19.33594	13
−6.3887	12.87032	83.46875	25	12.11112	33.39844	17
−7.0313	11.8302	86.13281	28	15.442	17.77344	21

**Figure 8:**
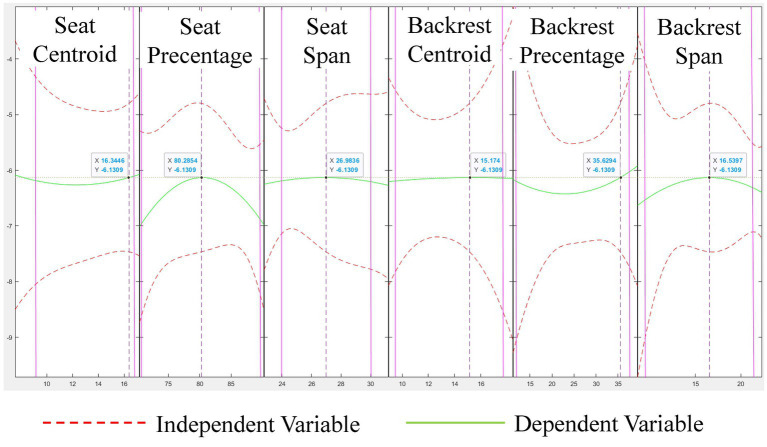
Multivariate nonlinear regression curve plot.

Firstly, import the data of 6 independent variables into a 20 × 6 matrix as input variables, denoted as $X=\left[x_{1},x_{2},x_{3},x_{4},x_{5},x_{6}\right]$ , and import the dependent variable into a 20 × 1 matrix as output, denoted as Y . Secondly, invoke the rstool command to import the input, output, and the model, as shown in Figure 8. The images from left to right represent the centroid coordinates of pressure points at the cushion, the percentage of pressure area at the cushion, the range of pressure span at the cushion, the centroid coordinates of pressure points at the backrest, the percentage of pressure area at the backrest and the range of pressure span at the backrest and the regression relationship between the percentage decline of MPF. The red dotted line represents the independent variable, the green represents the dependent variable, and the purple line represents the change interval of the actual data.

## 4 Result and discussion

By moving the data label in Figure 8, it can be seen that when the centroid row coordinate of the cushion pressure point is about 16, the cushion pressure area percentage is about 80%, and the cushion pressure span range is about 27, the backrest pressure point centroid row coordinate is about 15, the backrest pressure area percentage is about 35%, and the backrest pressure span range is about 16. At this time, the MPF value of the subjects decreased by a small percentage, and the fatigue development of the muscles was slower. In addition, we can see that the pressure area percentage at the seat cushion is a more sensitive independent variable, too large or too small pressure area percentage will easily cause lumbar muscle fatigue, and too small pressure area percentage is caused by the seat position too far forward or the big legs do not touch the seat cushion. The excessive pressure area percentage is caused by the seating position too far back, which is also confirmed by the changing trend of the pressure point centroid coordinates at the cushion and the pressure span range at the cushion. In addition, the data analysis results of the backrest can show that maintaining a large percentage of pressure area is conducive to delaying the generation of muscle fatigue, that is, people should contact the back of the wheelchair as much as possible to reduce the burden on the waist, whether it is the back or the waist to contact the back of the wheelchair can play a certain effect, depending on the individual’s sitting habits. However, try to avoid the sitting posture with the chest, maintaining such a sitting posture for a long time is easy to leads to fatigue of the waist muscles, which can be seen from the centroid coordinates of the pressure point at the back.

Due to the limitations of the site and experimental conditions, the experimental subjects in this paper are all men. Considering the differences in body structure between different genders, the experimental results of women may be somewhat deviated from the current results. Therefore, we plan to recruit female volunteers to further supplement the experimental samples in the future to improve the universality of the conclusions.

## 5 Conclusion

Because of the situation that wheelchair users are prone to lumbar muscle fatigue when they keep sitting for a long time, this paper hopes to study the relationship between different sitting positions and the development of lumbar muscle fatigue in the process of using wheelchairs, to provide more scientific guidance and suggestions for the use of wheelchairs. First, sEMG signal data from the upright spine muscle of the human body were collected, and the MPF decline percentage of the sEMG signal was obtained as the dependent variable through frequency domain analysis. Then, an array of thin film pressure sensors was used to obtain information on human sitting posture, and six features of pressure point centroid, area percentage, and span from the seat cushion and backrest were extracted as independent variables. Multivariate nonlinear regression is used to capture the relationship between independent variables and dependent variables. Through the analysis of the experimental data, we give the following suggestions for the use of wheelchairs: When riding a wheelchair, people should sit in the middle and back of the seat cushion, so that the Angle of the hip joint can be in a natural state, and the thigh part should fully contact the seat cushion to avoid the weight of the body concentrated on the hip; The back should also be fully in contact with the back of the wheelchair to reduce the burden on the waist, and the spine posture can be adjusted appropriately according to personal habits, but to avoid maintaining a chest for a long time, which will lead to the lumbar spine in an unnatural physiological Angle and easily lead to fatigue of the waist muscles.

## Data availability statement

The original contributions presented in the study are included in the article/supplementary material, further inquiries can be directed to the corresponding author.

## Author contributions

ZH: Methodology, Writing – original draft, Writing – review & editing. JC: Conceptualization, Funding acquisition, Methodology, Resources, Supervision, Writing – review & editing. YW: Conceptualization, Data curation, Formal analysis, Methodology, Writing – review & editing. SY: Validation, Visualization, Writing – review & editing.
